# Nutritional adequacy and dietary disparities in an adult Caribbean population of African descent with a high burden of diabetes and cardiovascular disease

**DOI:** 10.1002/fsn3.1363

**Published:** 2020-02-05

**Authors:** Rachel M. Harris, Angela M. C. Rose, Nita G. Forouhi, Nigel Unwin

**Affiliations:** ^1^ The George Alleyne Chronic Disease Research Centre Caribbean Institute for Health Research The University of the West Indies Bridgetown Barbados; ^2^ Faculty of Medical Sciences The University of the West Indies Cave Hill Barbados; ^3^ MRC Epidemiology Unit University of Cambridge Cambridge UK

**Keywords:** Barbados, diet, nutritional adequacy, sugar‐sweetened beverages

## Abstract

The Caribbean island of Barbados has a high burden of diabetes and cardiovascular disease. Dietary habits were last described in 2005. A representative population‐based sample (*n* = 363, aged 25–64 years) provided two nonconsecutive 24‐hr dietary recalls in this cross‐sectional study. Mean daily nutrient intakes were compared with the Dietary Guidelines for Americans. Subgroup differences by age, sex, and educational level were examined using logistic regression. High sugar intakes exist for both sexes with 24% (95% CIs 18.9, 30.0) consuming less than the recommended <10% of energy from added sugars (men 22%; 15.0, 31.6; women 26%; 18.9, 33.7). Sugar‐sweetened beverages provide 43% (42.2%, 44.4%) of total sugar intake. Inadequate dietary fiber intakes (men 21 g, 18.2, 22.8; women 18 g, 16.7, 18.9) exist across all age groups. Inadequate micronutrient intake was found in women for calcium, folate, thiamine, zinc, and iron. Older persons (aged 45–64 years) were more likely to report adequacy of dietary fiber (OR = 2.7, 1.5, 4.8) and iron (OR = 3.0, 1.7, 5.3) than younger persons (aged 25–44). Older persons (aged 45–64 years) were less likely to have an adequate supply of riboflavin (OR = 0.4, 0.2, 0.6) than younger persons. Men were more likely to have adequate intakes of iron (OR = 13.0, 6.1, 28.2), folate (OR = 2.4, 1.3, 4.6), and thiamine (OR = 3.0, 1.5, 5.0) than women. Education was not associated with nutrient intake. The Barbadian diet is characterized by high sugar intakes and inadequate dietary fiber; a nutrient profile associated with an increased risk of obesity, type 2 diabetes, and related noncommunicable diseases.

## Graphical abstract

The Caribbean island of Barbados has a high burden of diabetes and cardiovascular disease. The Barbadian diet is characterized by high sugar intakes and inadequate dietary fibre; a nutrient profile associated with an increased risk of obesity, type 2 diabetes, and related noncommunicable diseases.

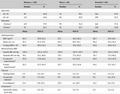

## 1 INTRODUCTION

Barbados is a small island developing state and the most easterly island of the Caribbean chain. The population is approximately 280,000; 94% being of African origin (Barbados Statistical Service, 2010). For Barbadians, aged between 30 and 70 years, women have a one‐in‐eight, and men a one‐in‐five, probability of dying from an NCD (Health Situation analysis, n.d.). Cardiovascular disease (CVD), primarily heart attack and stroke, type 2 diabetes and cancers are the leading causes of premature death in Barbados (IHME, 2013).The island's annual mortality rate from CVD is estimated at 275.6 per 100,000 with the annual years of life lost being approximated at 4,759 per 100,000 (“Cardiovascular Diseases in Barbados,” n.d.).The prevalence of type 2 diabetes for Barbados is estimated at 18.7% (95% CIs 16.2%; 21.4%) (Howitt et al., 2015), which is double the global figure. Barbados and the wider Caribbean have higher rates of premature NCD mortality than in other parts of the Americas (Ordunez, Prieto‐Lara, Pinheiro Gawryszewski, Hennis, & Cooper, 2015).

A suboptimal diet is a known major contributor to the development of obesity, type 2 diabetes, and CVD (Ezzati, Pearson‐Stuttard, Bennett, & Mathers, 2018) and also to prevalent micronutrient deficiencies. In Barbados, for example, it has been estimated that one‐in‐five women of reproductive age were anemic (2018 Global Nutrition Report, 2018). Poor dietary habits are responsible for more deaths globally than any other risk factor (GBD 2017 Diet Collaborators, 2019). Nutrition programmes are increasingly being integrated into public health policies with the focus now shifting away from the excessive intakes of unhealthy nutrients (Bruins, Van Dael, & Eggersdorfer, 2019) to the optimization of adequate nutrient‐rich diets (WHO Global Action Plan for the Prevention and Control of NCDs 2013–2020, n.d.). The Global Burden of Disease (GBD) Study estimates that a suboptimal diet contributes approximately 15% of total disability‐adjusted life years (DALYs) among adults worldwide (GBD 2017 Diet Collaborators, 2019). In 2017, more than half of diet‐related deaths and two‐thirds of diet‐related DALYs were attributable to high intakes of sodium, low intakes of whole grains, and low intakes of fruits. For the Caribbean, the GBD study estimated suboptimal intakes of fruits, vegetables, whole grains, nuts, and seeds, with inadequate levels of fiber and calcium (GBD 2017 Diet Collaborators, 2019). The estimates from the GBD study are in fact based on highly limited data from the region and rely on modeling and extrapolation. Dietary habits for Barbadians were last described in 2005 where it was noted that Barbados like many other countries of the world was undergoing a “nutrition transition” (Sharma et al., 2008). However, the data from this small study (*n* = 49) were based on a cancer case–control study and were not designed to be representative of the general adult population.

In 2012–2013, we conducted the population‐based Barbados Salt Study in adults aged 25–64 years, which included 24‐hr dietary recalls and urine collection. We have previously published the data on urine sodium and potassium excretion from this study (Harris et al., 2018). Here, we describe data on nutritional adequacy from this population, the most detailed account to date and including for the first time for this population analyses by social group. We aimed, therefore, to assess and describe dietary adequacy for Barbadians and to investigate the influence of age, sex, and educational level on nutrient intake. We anticipate that this work will add to an area with the current paucity of dietary data for the Caribbean region. These findings will be of importance for the formulation of effective public health policy and culturally appropriate nutritional interventions, targeting those groups at greatest risk, for the Barbadian population.

## 2 MATERIALS AND METHODS

### 2.1 Study population

The study population was a subsample of the Barbados Health of the Nation (HotN)Study, a cross‐sectional survey conducted in 2011–2013, which recruited a nationally representative sample of adults aged ≥25 years (*n* = 1,234). Details of the sampling, recruitment, and data collection methods have been published elsewhere (Harris et al., 2018; Howitt et al., 2015). A sample of 441 adults aged 25–64 years was selected, stratified by sex and age group (25–44 and 45–64 years), with the aim of recruiting at least 100 persons in each group. The methods used to collect biochemical (cholesterol and blood glucose), anthropometric, behavioral, and medical history data have been previously described (Howitt et al., 2015). Information on diet was collected during two face‐to‐face interviews carried out in 2012–2013. Data on diabetes, hypertension, and cholesterol were collected in the main HotN study (Howitt et al., 2015). Diabetes was defined by self‐reported doctor‐diagnosed diabetes or fasting plasma glucose ≥7 mmol/L while hypertension was defined by self‐reported current use of antihypertensive medication, or systolic blood pressure ≥140 mm Hg or a diastolic blood pressure ≥90 mm Hg. Pregnant and lactating women were excluded because of their unique nutritional requirements. All procedures followed were in accordance with the Helsinki Declaration of 1975 as revised in 1983, with ethical approval granted by the Institutional Review Board of the Ministry of Health, Barbados and the University of the West Indies. All participants provided written informed consent.

### 2.2 Procedures

Two nonconsecutive, interviewer‐administered, 24‐hr dietary recalls were collected from each participant by trained data collectors, using the United States Department of Agriculture (USDA) multi‐pass method. The 24‐hr dietary recall is considered to be the gold standard method for assessing mean intake at the population level (GBD 2017 Diet Collaborators, 2019). Portion size consumed in one sitting was captured using three‐dimensional Nasco food models (Nasco Company), standard measuring cups, and household utensils. The timing, food source, frequency of consumption, cooking method, seasoning use, and recipes were documented.

#### 2.2.1 Dietary data analysis

Individual food items were coded, and recorded portions converted into grams by a Registered Dietitian (author RH). These data were double entered, into the nutrition software, Nutribase Pro (version 9, Cybersoft Inc.). The underlying databases for this program are the USDA and Canadian food composition tables. The Association of Official Agricultural Chemists (AOAC) method was used to estimate the fiber levels in foods included in these food composition tables (Nutribase 9 User's Manual, 2010). Standardized traditional Barbadian recipes (Sharma et al., 2007) were added to the underlying food composition database, making this software more culturally appropriate.

#### 2.2.2 Food grouping

Foods were placed into food categories guided by previous work done in Barbados (Sharma et al., 2008). All food categories were reviewed by a Registered Dietitian (author RH). Food groups were combined as follows: “vegetables” consisted of all fresh, frozen, and canned vegetables, but not including legumes and potatoes; “fish” comprised fatty and lean fish, crustaceans and molluscs; “red meat” included all red meat including processed canned products, organ, and minced meat; and “poultry” included all chicken and turkey meats and their products. “Fruit” consisted of whole fruit including fresh, frozen, and canned fruits. “Sugar‐sweetened beverages (SSBs)” consisted of sweetened juices, homemade juices with added sugar, juice drinks, fruit flavored drinks made from sugar crystals and flavored milks, sodas or carbonated drinks, sports and energy drinks, and any sweetened hot drinks (e.g., hot chocolate and coffee). “Beverages with no added sugar” included 100% pure fruit juice, smoothies, diet sodas, and unsweetened tea and coffee. “Ground provisions” included sweet potato, yams, cassava, eddoes, green bananas, breadfruit, plantains, English potato, and French fries; “dairy” included milk, yogurt, cheese, ice‐cream, butter, and cream. Cereals were split into two broad categories, “hot porridges” and “other cereals”; breads were also divided into two groups, “multigrain and bran” and “white”; “nuts and legumes” included all tree nuts, peanuts, nut butters, and legumes; “rice” and “pasta” included both refined and unrefined products as well as all composite dishes, such as macaroni pie and rice and peas. “Cakes and sweetbreads” included all baked products and cookies; “chocolate drinks” included all chocolate flavored water‐based beverages, “candy/sweets, chocolate” included all sweets, candy, mints, and chocolates; “sugar” included all types of sugar, honey, syrups, jams, and jellies.

The 2015–2020 Dietary Guidelines for Americans defines added sugars as “syrups and other caloric sweeteners used as a sweetener in other food products” (USDA, Dietary Guidelines for Americans, 2015–2020).This does not include naturally occurring free sugars such as in fruit (fructose) and milk (lactose). For this work reported total sugar (g/day) is comprised of, monosaccharides (glucose, fructose, and galactose) and disaccharides (sucrose, maltose, and lactose) originating from free sugars including added sugar sources.

The percentage contribution made by each food category either in terms of calories for energy or by weight in grams for fat, protein, total sugar, and fiber were calculated. These percentages were then ranked and the “top 10” contributing food categories for each macronutrient and dietary fiber determined.

#### 2.2.3 Socioeconomic indicators and age

Data on education were collected by self‐report as the highest level of education completed. The data were available for almost all study participants and education was therefore chosen as the measure of socioeconomic status. Due to the small sample size (*n* = 364), education was divided into less than tertiary education and tertiary education. Tertiary education was defined as postsecondary education including college, vocational, and university training. Age was grouped into older (45–64 years) and younger (25–44 years) age groups.

### 2.3 Statistical analyses

All analyses were weighted to account for the sampling design, nonresponse and to match the age–sex distribution of the Barbadian population according to the 2010 Barbados Population and Housing Census (Barbados Statistical Service, 2010). We excluded dietary recalls from all statistical analyses where the energy intake was extreme being >4,780 kcal/day for men and >4,302 kcal/day for women and <500 kcal/day (Bradbury, Tong, & Key, 2017). All continuous variables approximated a normal distribution therefore means or proportions (as appropriate) are presented, with 95% confidence intervals (CI) for both macronutrient and specific micronutrients. To obtain the mean individual daily intake for specific nutrients, the sum total for each nutrient recorded for the two recall days was divided by 2 (Sharma et al., 2008).

The assessment of nutritional adequacy involves the comparison of recommended with estimated nutrient intakes. Food‐based dietary guidelines do exist for Barbados; however, no specific nutrient targets are outlined (Food based dietary guidelines for Barbados, 2017). In the absence of up to date Caribbean nutritional guidelines (last updated in 1994) (CFNI: Recommended Dietary Allowances for the Caribbean), we used the 2015–2020 Dietary Guidelines for Americans (DGA) (USDA, Dietary Guidelines for Americans, 2015–2020) (Table S1c) which include nutritional goals for age–sex groups based on dietary reference intakes as the reference values to assess nutritional adequacy. The prevalence of nutrient inadequacy was assessed using the Recommended Dietary Allowance (RDA) cut‐point method where the proportion of men and women meeting the recommendation was determined.

We performed logistic regression to investigate the association by each of age, sex, and educational level on nutrient adequacy, while controlling for the other two as potential confounder. Statistical significance is reported as *p* < .05. We provide the odds ratios (ORs) and their associated 95% confidence intervals for dietary variables only for which statistical significance was found at *p* < .05 (i.e., where the ORs do not cross 1). Data analysis was performed using STATA v12 (StataCorp. LP, College Station, TX, USA). All estimates and analyses were adjusted using the “SVY” module of STATA v12 to account for the complex survey sampling design.

## 3 RESULTS

The study population demographic and health characteristics are shown in Table 1. The overall prevalence of obesity (BMI ≥ 30) was 36.0%, being almost twice as high in women (46.4%) than men (25.3%). The prevalence of hypertension (defined by a systolic blood pressure >140 mm Hg or a diastolic blood pressure ≥90 mm Hg) and diabetes was 34.1% and 13.1%, respectively, being similar for both sexes. The mean total‐to‐HDL cholesterol ratio was higher in men (6.5) than women (5.4) (Table 1).

**Table 1:** Demographic and health characteristics of the Barbados National Salt Study (BNSS) sample by age, sex, and educational level (2012–2013)

	Women *n* = 203		Men *n* = 161		Overall *N* = 364	
	Number	%	Number	%	Number	%
Age years						
25–44	90	46.0	76	49.1	166	47.8
45–64	113	54.0	85	50.9	198	52.2
Education						
<Tertiary^a^	127	77.9	99	76.3	226	77.2
Tertiary^a^	76	22.1	62	23.7	138	22.8

	Mean	95% CI	Mean	95% CI	Mean	95% CI
Anthropometry						
BMI, kg/m^2^	30.3	29.0, 31.6	27.1	26.0, 28.2	28.7	27.9, 29.6
Waist circ, cm	94.7	91.9, 97.4	92.4	89.7, 95.1	93.6	91.9, 95.2
% obese BMI ≥ 30	46.4	38.4, 54.6	25.3	19.0, 32.8	36.0	30.8, 41.5
Blood pressure (BP)						
Systolic BP, mm Hg	124.4	121.6, 127.2	130.5	127.4, 133.7	127.3	125.3, 129.4
Diastolic BP, mm Hg	78.1	76.1, 80.0	79.5	77.1, 81.8	78.7	77.3, 80.2
% reported hypertension^b^	25.8	17.8, 36.0	14.4	9.6, 21.0	20.4	15.5, 26.3
% total hypertension^b^	35.5	27.5, 44.4	32.7	23.2, 43.8	34.1	27.7, 41.2
Glucose						
Fasting/mmol	5.5	5.2, 5.8	5.4	5.2, 5.6	5.4	5.3, 5.6
% reported diabetes^c^	9.9	5.7, 16.5	9.0	5.0, 15.6	9.4	6.6, 13.3
% all diabetes^c^	15.1	9.8, 22.5	10.9	6.1, 18.7	13.1	9.6, 17.6
Cholesterol						
Total/HDL cholesterol ratio	5.4	5.0, 5.8	6.5	5.9, 7.0	5.9	5.5, 6.2

a Tertiary education was defined as postsecondary education including college, vocational, and university training.

b Hypertension was defined as self‐reported current use of antihypertensive medication, or a systolic blood pressure ≥140 mm Hg, or a diastolic blood pressure ≥90 mm Hg.

c Diabetes was defined as self‐reported doctor‐diagnosed diabetes or fasting blood glucose ≥7 mmol/L.

A total of 722 days of dietary data were analyzed. More weekdays (75%) than weekend days (25%) were captured, and approximately 15% of persons reported to be on a “special diet”. The nutrient intakes by age and sex are shown in Table S1a. The estimated energy intakes were higher in men (2,333 kcal/day; 2,172.4, 2,494.0) than women (1,840 kcal/day; 1,734.5, 1,946.4). The mean percentage energy from carbohydrate (53%), protein (17%), and fat (28%–30%) were similar for both sexes and across all age groups. The mean intake of total sugars was 127 g/day (117.7, 136.3) for men and 99 g/day (90.9, 106.9) for women. The intake of the essential fatty acids, linoleic (men 4 g; 3.4, 4.8; women 4 g; 3.3, 4.2), and linolenic acids (men 0.36 g; 0.25, 0.46; women 0.36 g; 0.29, 0.43) were similar for both sexes. The intake of dietary fiber was low (men 21 g; 18.2, 22.8; women 18 g; 16.7, 18.9).

Estimated micronutrient intakes were all higher in men than women (Table S1b). Men 25–44 years had the highest intakes of vitamin B6 (2.2 mg; 1.7, 2.7), calcium (739 mg; 656.3, 821.4), iron (16 mg; 13.3, 18.3), zinc (10 mg; 9.2, 11.5), and vitamin B12 (24 µg; 18.0, 30.4). Men (45–64 years) had the highest intake of folate (356 µg; 304.2, 407.7) and vitamin C (148 mg; 120.9, 175.7). The lowest intakes of folate (273 µg; 250.2, 295.2) and vitamin C (109 mg; 90.0, 127.2) were found in women aged 45–64 years.

Macronutrient adequacy is reported in Table 2. Over 80% of persons for both sexes met the RDA for protein. Approximately 70% were within the Acceptable Macronutrient Distribution Range (AMDR) of 20%–35% energy from fat. More women (71%; 63.0, 77.4) than men (64%; 57.4, 70.4) fell within the AMDR of 45%–64% energy from carbohydrate. Dietary fiber intakes were inadequate, for both sexes and across all age groups (men 19%; 12.9, 27.8; women 15%; 10.3, 22.2). Only 24% (18.9, 30.0) of persons consumed below the recommended <10% of energy from added sugars (men 22%; 15.0, 31.6; women 26%; 18.9, 33.7).

**Table 2:** Proportion of adult men and women (25–64 years) in Barbados meeting the 2015–2020 Dietary Guidelines for Americans^a^ for macronutrients (2012–2013)

Nutrient, unit	Men:% meeting RDA (95% CI)	Women: % meeting RDA (95% CI)	Overall: % meeting RDA (95% CI)
Carbohydrate, g	99 (94.7, 99.8)	96 (90.7, 98.4)	97 (94.6, 98.7)
Protein, %kcal	93 (86.6, 96.8)	94 (89.0, 96.5)	94 (89.6, 96.11)
Protein, g	84 (74.1, 90.2)	86 (77.9, 91.0)	85 (79.1, 89.0)
Saturated fat, %kcal	80 (72.2, 86.3)	71 (61.6, 78.2)	75 (69.4, 80.1)
Total fat, %kcal	70 (62.1, 76.7)	71 (64.1, 77.6)	71 (65.4, 75.5)
Carbohydrate, %E	64 (57.4, 70.4)	71 (63.0, 77.4)	68 (62.4, 72.4)
Dietary fiber, g	19 (12.9, 27.8)	15 (10.3, 22.2)	17 (13.4, 21.9)
Added sugars, %kcal	22 (15.0, 31.6)	26 (18.9, 33.7)	24 (18.9, 30.0)

a Table S1c**: **Daily nutritional goals for age‐sex groups based on dietary reference intakes and dietary guidelines for Americans 2015‐2020.

Inadequate intakes of calcium, folate, and zinc were observed for both sexes, while iron was inadequate for women only (39%; 31.7, 47.7). Intakes of thiamine (37%; 30.7, 42.8), riboflavin (40%; 33.0, 47.1), and vitamin B6 (46%; 39.7, 52.3) were all lower than recommended. Approximately half of the population met the RDA for vitamin C (60%; 53.8, 66.2) (Table S1b and Table 3).

**Table 3:** Proportion of adult men and women (25–64 years) in Barbados meeting the 2015–2020 Dietary Guidelines for Americans^a^ for specific micronutrients (2012–2013)

Nutrient, unit	Men: % meeting RDA (95% CI)	Women: % meeting RDA (95% CI)	Overall: % meeting RDA (95% CI)
Vitamin B12, µg	92 (83.7, 95.9)	92 (86.1, 95.8)	92 (87.3, 95.0)
Iron, mg	88 (79.8, 93.1)	39 (31.7, 47.7)	62 (54.7, 69.6)
Vitamin C, mg	60 (51.9, 68.1)	60 (52.1, 67.5)	60 (53.8, 66.2)
Vitamin B6, mg	53 (44.1, 62.0)	39.0 (30.9, 48.6)	46 (39.7, 52.3)
Riboflavin (B2), mg	46 (36.2, 55.5)	34 (26.3, 43.7)	40 (33.0, 47.1)
Thiamine (B1), mg	48 (37.5, 59.4)	26 (19.6, 33.3)	37 (30.7, 42.8)
Zinc, mg	32 (24.1, 41.7)	37 (28.6, 45.3)	35 (28.8, 40.7)
Total folate, µg	33 (24.0, 43.1)	17 (11.5, 23.9)	24 (19.3, 30.4)
Calcium, mg	19 (12.7, 26.6)	9 (3.9, 19.2)	14 (9.2, 19.4)

a Table S1c: Daily nutritional goals for age‐sex groups based on dietary reference intakes and dietary guidelines for Americans 2015–2020.

Results of logistic regression for only those nutrients found to have statistically significant associations (*p *= <0.05) with age, sex, or educational level are shown in Table 4. Older persons (aged 45–64 years) were more likely to report adequacy of dietary fiber (OR = 2.7, 1.5, 4.8) and iron (OR = 3.0, 1.7, 5.3) than younger persons (aged 25–44). Older persons (aged 45–64 years) were less likely to have an adequate supply of riboflavin (OR = 0.4, 0.2, 0.6) than younger persons. Men were more likely to have adequate intakes of iron (OR = 13.0, 6.1, 28.2), folate (OR = 2.4, 1.3, 4.6), and thiamine (OR = 3.0, 1.5, 5.0) than women. For this population, age and sex were more strongly associated with nutrient intake than education, with women generally being at greater risk of dietary inadequacy than men. Persons of a higher level of education were less likely to have an adequate intake calcium compared with persons of a lower educational level (OR = 0.4, 0.1, 1.0).

**Table 4:** Age, sex, and educational level^a^, ^a^ as predictors of nutrient adequacy, in logistic regression

Nutrient	OR	95% CI	*p* value
Dietary fiber			
Older versus younger	2.7	1.5, 4.8	.001^a^, *
Sex (men vs. women)	1.3	0.6, 2.8	.437
Education (higher vs. lower)	1.8	1.0, 3.4	.062
Folate			
Older versus younger	0.7	0.4, 1.2	.019^a^, *
Sex (men vs. women)	2.4	1.3, 4.6	.008^a^, *
Education (higher vs. lower)	1.5	0.9, 2.4	.123
Iron			
Older versus younger	3.0	1.7, 5.3	.001^a^, *
Sex (men vs. women)	13.0	6.1, 28.2	<.001^a^, *
Education (higher vs. lower)	1.2	0.7, 2.0	.539
Thiamine			
Older versus younger	0.7	0.4, 1.1	.108
Sex (men vs. women)	3.0	1.5, 5.0	.002^a^, *
Education (higher vs. lower)	0.6	0.4, 1.0	.058
Calcium			
Older versus younger	0.6	0.3, 1.1	.074
Sex (men vs. women)	2.4	0.9, 6.5	.093
Education (higher vs. lower)	0.4	0.1, 1.0	.046^a^, *
Riboflavin			
Older versus younger	0.4	0.2, 0.6	.000^a^, *
Sex (men vs. women)	1.6	1.0, 2.7	.072
Education (higher vs. lower)	0.9	0.4, 1.8	.670

a Analyses by age, sex and educational level entered together.

* Statistical significance taking the other 2 variables into account.

The top 10 food sources for energy, protein, fat, total sugar, and dietary fiber are shown in Table 5. The top three food sources for energy (SSBs, poultry, and ground provisions), protein (poultry, fish, and red meat), and fat (poultry, red meat, and dairy) collectively made up 28%, 48%, and 33% of each, respectively. Sources of healthier fats, such as fish and nuts, contributed a much smaller proportion to total fat (12%). The top source of total sugar was SSBs (43%), which provides approximately 54% of added sugar in the Barbadian diet. The top three sources of dietary fiber (ground provisions, bread, and fruit) provided approximately 43% of total dietary fiber.

**Table 5:** The ten major food sources for energy, protein, fat, sugar, and fiber from the 24‐hr dietary recalls, in adults 25–64 years of age in Barbados (2012–2013)

Food sources of total energy intake	% contribution to energy (kcal) (95% CI)	Food sources of protein	% contribution to total protein (g) (95% CI)	Food sources of total fat	% contribution to total fat (g) (95% CI)	Food sources of sugar	% contribution to total sugar (g) (95% CI)	Food sources of dietary fiber	% contribution to fiber (g) (95% CI)
Sugar‐sweetened beverages	9.99 (9.35, 10.63)	All poultry	26.00 (25.06, 26.95)	All poultry	14.13 (13.38, 14.88)	Sugar‐sweetened beverages	43.29 (42.23, 44.35)	Ground provisions	17.14 (16.33, 17.95)
All poultry	9.58 (8.95, 10.21)	Fish	11.30 (10.62, 11.98)	Red Meat	10.83 (10.16, 11.49)	Fruit	11.25 (10.57, 11.93)	Bread	14.02 (13.28, 14.77)
Ground provisions	8.71 (8.10, 9.31)	Red Meat	10.76 (10.09, 11.42)	Dairy	8.06 (7.48, 8.65)	Cakes, sweetbreads	6.80 (6.26, 7.34)	Fruit	12.26 (11.56, 12.96)
Rice	8.64 (8.04, 9.24)	Bread	7.44 (6.88, 8.00)	Cakes, sweetbreads	7.95 (7.37, 8.53)	Dairy	6.10 (5.58, 6.61)	Vegetables, ± salt	10.51 (9.85, 11.17)
Bread	8.20 (7.61, 8.79)	Pasta	5.76 (5.26, 6.26)	Ground provisions	7.21 (6.66, 7.77)	Ground provisions	3.91 (3.50, 4.33)	Rice	8.31 (7.72, 8.90)
Cakes, sweetbreads	5.86 (5.36, 6.36)	Processed meats	5.59 (5.10, 6.08)	All fish	6.31 (5.78, 6.83)	Bread	3.74 (3.33, 4.15)	Cereals	7.08 (6.53, 7.63)
Pasta	5.81 (5.31, 6.32)	Dairy	5.48 (5.00, 5.97)	Pasta	6.25 (5.73, 6.77)	Candy/sweets, chocolate	3.65 (3.25, 4.05)	Cakes, sweetbreads	4.42 (3.98, 4.86)
Red Meat	5.44 (4.95, 5.93)	Rice	5.27 (4.79, 5.74)	Processed meats	6.09 (5.58, 6.61)	Beverages with no added sugar	3.29 (2.91, 3.67)	Nuts, legumes	3.86 (3.44, 4.27)
Dairy	4.65 (4.20, 5.10)	Ground provisions	3.56 (3.17, 3.96)	Nuts, legumes	5.35 (4.87, 5.83)	Vegetables, ± salt	2.56 (2.22, 2.90)	Pasta	3.64 (3.24, 4.05)
Fish	4.43 (3.98, 4.87)	Cereals	2.31 (1.98, 2.63)	Bread	4.37 (3.93, 4.81)	Cereals	2.06 (1.76, 2.36)	Crackers	3.35 (2.97, 3.74)
Total %	71.31%		83.47%		76.55%		86.65%		84.59%

## 4 DISCUSSION

The current study highlights several dietary factors with discordance between the recommendations and reported intakes in a representative national sample of adults in Barbados. According to this study, the nutrient profile for Barbadians is characterized by high sugar intakes, with half of total sugar intake coming from added sugars and half of added sugars coming from SSBs. Moreover, the intakes of dietary fiber were inadequate. For this population, age and sex appear to be stronger determinants of dietary intake than education, with women generally being at greater risk of dietary inadequacy than men. We focus our discussion around those results which we consider to be of public health importance.

The reduction of the current level of consumption for SSBs (10% of total estimated daily calories) in Barbados should be an urgent public health priority. SSBs are energy‐dense beverages of poor nutritional value which have been associated with an increased risk of obesity and type 2 diabetes (Fagherazzi et al., 2013) and a greater prevalence of dyslipidaemia (Malik, Popkin, Bray, Després, & Hu, 2010). Each serving/d of habitual intake of SSBs has been associated with an 18% greater incidence of type 2 diabetes (Imamura et al., 2016) and a modeling study performed by the Global Burden of Diseases Nutrition and Chronic Diseases Expert Group (NutriCoDE) estimated that up to 184,000 deaths per year could be attributed to the chronic overconsumption of SSBs (Fagherazzi et al., 2013). The WHO recommends that governments introduce taxation targeting sugary drinks (Popkin & Hawkes, 2016; Summary report on the technical consultation on reducing sugar intake in the Eastern Meditteranean Region. 2015, n.d.), in an effort to alter consumer behavior and incentivize product reformulation (Baker, Jones, & Marie Thow, 2017). In Mexico, the implementation of an excise tax of 10% to all nonalcoholic beverages with added sugar which provided an estimated 9.8% of total calories resulted in an average 6.1% reduction in SSBs consumption. (Barrientos‐Gutierrez et al., 2017). In September 2015, Barbados introduced a 10% excise tax on SSBs as an initial step toward reducing the overall population consumption (Alvarado et al., 2017). Evaluation of the effectiveness of this fiscal intervention in Barbados has found that weekly sales of SSBs reduced by 4.3% (Alvarado et al., 2019). Taxation initiatives were highlighted at the regional 38th Caribbean Community (CARICOM) Heads of Government Conference in 2017 as a way to reduce the consumption of foods high in salt, fat, and sugar including SSBs.

Globally, governments have also introduced measures to inform and increase public awareness of the potential dangers to health posed by excessive consumption of SSBs in the diet. The “Pouring on the Pounds” campaign introduced in the United States in 2009 used graphic slogans such as “Don't drink yourself fat” (Popkin & Hawkes, 2016) to deliver their message. Sugary drinks have also been targeted in public awareness campaigns in Australia as part of the “Live Lighter” campaign (About LiveLighter. About Sugary Drinks Western Australia: About LiveLighter, 2015), in the UK Change4Life social marketing campaign and in Tonga with the “A Mouthful of Sugar” campaign in 2012 (Popkin & Hawkes, 2016). Regionally in Jamaica, the “Are you drinking yourself sick?” campaign was launched in 2017 (Are You Drinking Yourself Sick? ‐ Heart Foundation, Health Ministry Launch, n.d.). For Barbados, initiatives are underway to increase public awareness of the dangers of overconsumption of SSBs. The Healthy Caribbean Coalition (HCC) is spearheading a campaign which aims to educate the public on the dangers of habitual consumption of SSBs while encouraging the increased consumption of water and beverages without added sugars. This study is timely in that it will provide baseline metrics which can be used in the development of culturally appropriate public health messages and policy for Barbadians. The influence of cultural factors such as the personal beliefs of Barbadians around SSBs is, however, an area yet to be explored.

The US average intake for dietary fiber is estimated at 15.7–17.0 g/day and 13.1 g/day for non‐Hispanic Blacks (Grooms, Ommerborn, Pham, Djoussé, & Clark, 2013), similar to estimated levels for Barbadians. An increase of dietary fiber in the diet is of paramount importance as it has been shown to be inversely associated with coronary heart disease (Wu et al., 2015) and stroke (Wang et al., 2014). Results from a recent meta‐analysis suggest that individuals who consume higher amounts of dietary fiber, especially cereal fiber, may benefit from a reduction in the incidence of developing type 2 diabetes (InterAct Consortium, 2015). A small reduction in fasting blood glucose concentration, as well as a small reduction in glycosylated hemoglobin percentage for individuals with type 2 diabetes who add β‐glucan or psyllium to their daily dietary intake, was also noted (McRae, 2018). Increased intakes of fruits, vegetables, whole grains, legumes, and ground provisions should be encouraged for this population, especially among younger age groups (25–44 years). The WHO has recommended governments introduce subsidies for fruits and vegetables (Summary report on the technical consultation on reducing sugar intake in the Eastern Meditteranean Region. 2015, n.d.). Barbados uses fiscal policy with the aim to make healthy foods more affordable. Currently in Barbados, a “basket of goods” which includes some fruits and vegetables is exempt from taxation (Barbados Revenue Authority, 2017).

The total‐to‐HDL cholesterol ratio (Atherogenic or Castelli Index) is above the ideal ratio of 3.5 recommended by the American Heart Association for both sexes. The predictive value of this ratio is known to be greater than its isolated parameters (Millán et al., 2009). This ratio is a powerful predictor of ischemic heart disease risk and has also been associated with insulin resistance syndrome (Lemieux et al., 2001). Fat intakes despite being at an acceptable level for Barbadians were mainly from sources which are high in saturated (poultry, red meat, and dairy products) and trans‐fat (cakes and sweetbreads). The promotion of healthier cardio‐protective monounsaturated fats, omega‐3, and omega‐6 fatty acids from foods such as oily fish, plant‐based oils (such as flaxseed, soybean, and canola oils) eggs and nuts should be encouraged, in an effort to reduce LDL‐cholesterol while preserving HDL cholesterol (Mensink, Zock, Kester, & Katan, 2003). Processed meats make a notable contribution (6%) toward total fat intake in Barbados. Red and processed meat consumption has been associated with the development of type 2 diabetes (Pan et al., 2013). Several additives found in processed meats such as nitrites and their by‐products (e.g., peroxynitrite) experimentally promote atherosclerosis and vascular dysfunction (Förstermann, 2008), reduce insulin secretion (Förstermann, 2008), and impair glucose tolerance (Portha, Giroix, Cros, & Picon, 1980). Alternative protein sources such as poultry, fish, and legumes should be encouraged.

Folate, vitamin B6, and vitamin B12 are involved in homocysteine metabolism, which are thought to reduce CVD risk by lowering homocysteine levels (Clarke et al., 2010). From this study, only 39% of women were estimated to consume enough iron. The RDA for iron, for women between 19 and 50 years (18 mg/day) is considerably higher than for women ≥50 years (8 mg/day). The intake of vitamin C which aids in the absorption of this mineral is low in women aged 45–64 years. These two factors may help to explain why the proportion of women estimated to consume adequate daily iron is low. Women, especially those of reproductive age (19–50 years), need to be targeted in future nutrition intervention programmes. Dietary supplementation or the greater inclusion of foods high in these nutrients, such as pulses, eggs, lean red meats, and leafy vegetables should be encouraged.

Limitations of this study include the cross‐sectional design which does not allow for temporal trends in the intake of foods and nutrients to be explored. The relatively small sample size may have limited our power to detect further significant associations between diet and age, sex and educational level. The *p*‐values attained for several nutrients (fiber, iron, thiamine, and riboflavin) investigating the association of education were close to the level of significance (*p* = .05). Perhaps if our sample size was larger, a significant difference may have been found.

Despite selecting two nonconsecutive days for dietary recalls, the fact that we collected dietary data mainly on weekdays may also have influenced our findings. In the absence of up to date Caribbean nutritional guidelines (last updated in 1994) (CFNI: The Caribbean Food & Nutrition Institute. Recommended Dietary Allowances for the Caribbean), we used the 2015–2020 Dietary Guidelines for Americans (DGA) (USDA, Dietary Guidelines for Americans, 2015–2020) (Table 1c) as the reference values to assess nutritional adequacy and this is a further limitation of our study. Dietary supplements were not included and accounted for in the nutrient outputs this may have led to an underestimation of actual nutrient intake. Study strengths include the use of a nationally representative sample for which weighting procedures were used to adjust for differences between the sample and population distributions, allowing our study findings to be generalized to the Barbadian population. All data were collected following internationally recognized standardized procedures. The dietary analysis programme Nutribase was made more culturally appropriate through the addition of 30 traditional commonly consumed Barbadian dishes. We do recognize, however, that for all dietary analysis programmes, nutrient intakes were best estimates only. For example, the consumption of sugar is likely to have been underestimated. Comparison of the nutrient content of some of the locally produced juices and carbonated beverages with similar products selected in Nutribase revealed a lower level of sugar in the Nutribase options. Further work is required in this area to more accurately assess the contribution made to overall diet by SSBs in Barbadian adults.

In conclusion, we have presented novel population‐based dietary data for Barbados. The Barbadian diet is characterized by high intakes of sugar and SSBs, with inadequate intake of fiber. Future work detailing the dietary patterns for this population is warranted as they may provide a better insight into relationships between nutrition and health. Our findings have identified areas of nutrition priority in Barbados and have provided valuable dietary data to the Caribbean region, highlighting the crucial need to improve overall diet in this population.

## CONFLICT OF INTEREST

The authors declare that they do not have any conflicts of interest.

## ETHICAL APPROVAL

Ethical Review: All procedures followed were in accordance with the Helsinki Declaration of 1975 as revised in 1983, with ethical approval granted by the Institutional Review Board of the Ministry of Health, Barbados and the University of the West Indies.

## INFORMED CONSENT

Written informed consent was obtained from all study participants.
