# Supplementary material for: Nutritional adequacy and dietary disparities in an adult Caribbean population of African descent with a high burden of diabetes and cardiovascular disease
Source: Food Sci Nutr. 2020 Feb 5;8(3):1335–44. doi: 10.1002/fsn3.1363 (PMC7063363; doi:10.1002/fsn3.1363)
Supplement: Supplementary file 1 [file FSN3-8-1335-s001.docx]

**Appendix 1:**

**Supplementary Table S1a:** Mean (95% confidence interval) daily energy and macronutrient intakes taken from 24-hour dietary recalls, by sex and age-group in adults 25–64 years of age in Barbados (2012–2013).

| **Nutrient, unit** | **Men** | | | **Women** | | |
| --- | --- | --- | --- | --- | --- | --- |
|  | **All ages**  **(95% CI)** | **25–44 years**  **(95% CI)** | **45–64 years**  **(95% CI)** | **All ages**  **(95% CI)** | **25–44 years**  **(95% CI)** | **45–64 years**  **(95% CI)** |
| **Energy, kcal/d** | 2333  (2172.4, 2494.0) | 2528  (2323.1, 2733.0) | 2107  (1908.1, 2305.6) | 1840  (1734.5, 1946.4) | 1899  (1723.6, 2074.7) | 1778  (1663.2, 1893.1) |
| **Carbohydrate, g** | 319  (296.7, 340.9) | 329  (301.3, 356.6) | 307  (276.5, 337.5) | 255  (240.7, 268.4) | 260  (37.7, 282.0) | 249  (230.7, 267.2) |
| **Carbohydrates, %E** | 53  (51.5, 54.6) | 51  (48.5, 53.0) | 56  (53.5, 57.8) | 53  (51.5, 54.5) | 53  (50.6, 54.5) | 54  (51.7, 55.4) |
| **Sugars, g** | 127  (117.7, 136.3) | 128  (115.0, 140.6) | 126  (107.4, 144.7) | 99  (90.9, 106.9) | 103  (90.5, 114.9) | 95  (83.5, 106.2) |
| **Protein, g** | 95  (86.7, 102.3) | 106  (95.9, 116.5) | 81  (71.6, 90.3) | 73  (66.7, 79.6) | 76  (65.0, 86.1) | 71  (65.6, 75.6) |
| **Protein, %E** | 17  (15.9, 17.7) | 17  (16.0, 18.5) | 16  (15.1, 17.3) | 17  (15.8, 17.5) | 17  (15.4, 17.7) | 17  (15.8, 17.7) |
| **Total Fat, g** | 72  (64.7, 78.4) | 81  (72.0, 89.9) | 61  (52.8, 68.5) | 60  (54.8, 64.3) | 62  (55.1, 69.1) | 57  (51.3, 62.3) |
| **Total Fat, % E** | 28  (26.4, 29.1) | 29  (27.3, 30.8) | 26  (24.3, 28.3) | 30  (28.6, 30.7) | 30  (28.8, 31.6) | 29  (27.4, 30.7) |
| **Saturated fat, g** | 21  (19.4, 23.2) | 24  (20.9, 26.4) | 18  (16.2, 20.7) | 17  (15.2, 18.6) | 18  (15.0, 20.5) | 16  (14.4, 17.6) |
| **Saturated fat, %E** | 8  (7.6, 8.6) | 8  (7.7, 9.0) | 8  (7.1, 8.5) | 8  (7.7, 8.6) | 8  (7.5, 9.1) | 8  (7.4, 8.6) |
| **Trans fats, g** | 0.22  (0.11, 0.33) | 0.26  (0.13, 0.39) | 0.18  (-0.01, 0.37) | 0.18  (0.11, 0.25) | 0.23  (0.11, 0.35) | 0.13  (0.05, 0.20) |
| **MUFA, g** | 21  (18.8, 24.1) | 25  (21.0, 28.2) | 18  (15.0, 20.7) | 17  (15.518.3) | 17  (15.2, 19.4) | 16  (15.0, 18.0) |
| **PUFA, g** | 14  (12.5, 15.6) | 16  (13.6, 18.0) | 12  (10.2, 13.7) | 11  (10.5, 12.3) | 12  (10.8, 13.4) | 11  (9.5, 11.8) |
| **Linolenic FA, g** | 0.36  (0.25, 0.46) | 0.42  (0.26, 0.58) | 0.28  (0.18, 0.38) | 0.36  (0.29, 0.43) | 0.37  (0.25, 0.50) | 0.35  (0.24, 0.45) |
| **Linoleic FA, g** | 4  (3.4, 4.8) | 5  (3.5, 5.7) | 4  (2.5, 4.5) | 4  (3.3, 4.2) | 4  (3.3, 4.6) | 3  (2.8, 4.1) |
| **Cholesterol, mg** | 256  (226.0, 285.4) | 290  (249.2, 330.2) | 216  (177.7, 254.7) | 232  (204.8, 258.5) | 233  (195.8, 270.3) | 230  (195.3, 265.1) |
| **Dietary fibre, g** | 21  (18.2, 22.8) | 20  (16.7, 23.7) | 21  (18.5, 23.3) | 18  (16.7, 18.9) | 16  (14.6, 17.6) | 20  (18.2, 20.9) |

**Supplementary Table S1b:** Mean micronutrient intakes by sex and age in adults 25-64 years of age in Barbados (2012-2013).

| **Nutrient, unit** |  | **Men** |  |  | **Women** |  |
| --- | --- | --- | --- | --- | --- | --- |
|  | **25-44 years**  **(95% CI)** | **45-64 years**  **(95% CI)** | **All ages**  **(95% CI)** | **25-44 years**  **(95% CI)** | **45-64 years**  **(95% CI)** | **All ages**  **(95% CI)** |
| **Vitamin C, mg** | 143  (114.7,170.5) | 148  (120.9,175.7) | 145  (124.1,166.3) | 127  (104.9,149.8) | 109  (90.0,127.2) | 118  (105.2,131.3) |
| **Vitamin B6, mg** | 2.2  (1.7,2.7) | 1.6  (1.3, 1.9) | 1.9  (1.6, 2.3) | 1.4  (1.7, 2.5) | 1.2  (1.0, 1.4) | 1.3  (1.1, 1.5) |
| **Total folate, µg** | 356  (304.2,407.7) | 359  (313.6, 403.4) | 357  (320.2,394.0) | 301  (251.2, 350.5) | 273  (250.2, 295.2) | 287  (260.4,314.0) |
| **Calcium, mg** | 739  (656.3,821.4) | 696  (606.9,784.9) | 719  (650.6,787.3) | 657  (533.0,780.1) | 593  (546.0,640.8) | 626  (558.8,693.0) |
| **Iron, mg** | 16  (13.3, 18.3) | 14  (12.2, 15.9) | 15  (13.3, 16.7) | 14  (11.2, 16.1) | 12  (11.2,13.6) | 13  (11.7, 14.4) |
| **Zinc, mg** | 10  (9.2, 11.5) | 9  (7.4, 9.6) | 9  (8.7, 10.3) | 7  (6.5, 8.1) | 7  (6.9,7.8) | 7  (6.8, 7.8) |
| **Vitamin B12, µg** | 24  (18.0, 30.4) | 20  (14.2, 25.7) | 22  (17.9, 26.5) | 23  (13.2, 32.4) | 15  (12.5,17.1) | 19  (13.7, 24.1) |

**Supplementary Table Ss1c:** Daily nutritional goals for age-sex groups based on dietary reference intakes and dietary guidelines for Americans 2015-2020.

| Sex  Age group(years) | Source of Goal | Female  19-30 | Male  19-30 | Female 31-50 | Male 31-50 | Female 51+ | Male 51+ |
| --- | --- | --- | --- | --- | --- | --- | --- |
| Calorie level(s) assessed |  | 2,000 | 2,400  2,600  3,000 | 1,800 | 2,200 | 1,600 | 2,000 |
| Protein, g | RDA | 46 | 56 | 46 | 56 | 46 | 56 |
| Protein, % kcal | AMDR | 10-35 | 10-35 | 10-35 | 10-35 | 10-35 | 10-35 |
| Carbohydrate, g | RDA | 130 | 130 | 130 | 130 | 130 | 130 |
| Carbohydrate, %kcal | AMDR | 45-64 | 45-64 | 45-64 | 45-64 | 45-64 | 45-64 |
| Dietary Fibre, g | 14g/1000kcal | 28 | 33.6 | 25.2 | 30.8 | 22.4 | 28 |
| % kcal Added sugars | DGA | <10 % | <10 % | <10 % | <10 % | <10 % | <10 % |
| Total fat, % kcal | AMDR | 20-35 | 20-35 | 20-35 | 20-35 | 20-35 | 20-35 |
| Saturated Fat, % kcal | DGA | <10 % | <10 % | <10 % | <10 % | <10 % | <10 % |
| Calcium, mg | RDA | 1000 | 1000 | 1000 | 1000 | 1200 | 1000 |
| Iron, mg | RDA | 18 | 8 | 18 | 8 | 8 | 8 |
| Zinc, mg | RDA | 8 | 11 | 8 | 11 | 8 | 11 |
| Vitamin C, mg | RDA | 75 | 90 | 75 | 90 | 75 | 90 |
| Vitamin B_6_, mg | RDA | 1.3 | 1.3 | 1.3 | 1.3 | 1.5 | 1.7 |
| Vitamin B_12_, mg | RDA | 2.4 | 2.4 | 2.4 | 2.4 | 2.4 | 2.4 |
| Folate, mcg, DFE | RDA | 400 | 400 | 400 | 400 | 400 | 400 |

*RDA = Recommended Dietary Allowance, AMDR = Acceptable Macronutrient Distribution Range, DGA = 2015-2020 Dietary Guidelines recommended limit.

Sources: Institute of Medicine. Dietary Reference Intakes: The essential guide to nutrient requirements. Washington (DC): The National Academies Press; 2006.

Institute of Medicine. Dietary Reference Intakes for Calcium and Vitamin D. Washington (DC): The National Academies Press; 2010
